# Decidual soluble factors participate in the control of HIV-1 infection at the maternofetal interface

**DOI:** 10.1186/1742-4690-8-58

**Published:** 2011-07-18

**Authors:** Romain Marlin, Marie-Thérèse Nugeyre, Marion Duriez, Claude Cannou, Anne Le Breton, Nadia Berkane, Françoise Barré-Sinoussi, Elisabeth Menu

**Affiliations:** 1 Regulation of Retroviral Infection Unit, Department of virology, Institut Pasteur, Paris, France; 2 Gynecology-Obstetrics Service, A. Béclère Hospital, AP-HP, Clamart, France; 3 Department of Gynecology Obstetrics and Reproductive Medecine, Tenon Hospital, AP-HP, UMPC, Paris, France

## Abstract

**Background:**

Maternofetal transmission (MFT) of HIV-1 is relatively rare during the first trimester of pregnancy despite the permissivity of placental cells for cell-to-cell HIV-1 infection. Invasive placental cells interact directly with decidual cells of the uterine mucosa during the first months of pregnancy, but the role of the decidua in the control of HIV-1 transmission is unknown.

**Results:**

We found that decidual mononuclear cells naturally produce low levels of IL-10, IL-12, IL-15, TNF-α, IFN-α, IFN-γ and CXCL-12 (SDF-1), and large amounts of CCL-2 (MCP1), CCL-3 (MIP-1α), CCL-4 (MIP-1β), CCL-5 (Rantes), CXCL-10 (IP-10), IL-6 and IL-8. CCL-3 and CCL-4 levels were significantly upregulated by *in vitro *infection with R5 HIV-1 but not X4. Decidual CD14+ antigen presenting cells were the main CCL-3 and CCL-4 producers among decidual leukocytes. R5 and X4 HIV-1 infection was inhibited by decidual cell culture supernatants *in vitro*. Using HIV-1 pseudotypes, we found that inhibition of the HIV-1 entry step was inhibited by decidual soluble factors.

**Conclusion:**

Our findings show that decidual innate immunity (soluble factors) is involved in the control of HIV-1 infection at the maternofetal interface. The decidua could thus serve as a mucosal model for identifying correlates of protection against HIV-1 infection.

## Background

Cytokines are involved in cell activation, immune response polarization and antiviral immunity, and play a key role in innate immunity. In particular, cytokines and chemokines can interfere with several steps of the Human Immunodeficiency Virus type 1 (HIV-1) replicative cycle. For instance, type 1 interferon (IFN) can induce the transcription of more than 100 genes, such as *Mx1*, *OAS *or *TRIM5α*, thereby inhibiting reverse transcription [1] and provirus integration [2]. Some chemokines inhibit HIV-1 entry by competitive binding to viral co-receptors [3,4]: CCL-3, CCL-4 and CCL-5 interact with the CCR5 co-receptor, thereby inhibiting the entry of R5 HIV-1, while CXCL-12 binds to CXCR4 and thus inhibits X4 HIV-1 entry. In contrast, proinflammatory cytokines such as IL-6, IL-12 and TNF-α stimulate HIV-1 replication by promoting inflammation or proviral genome transcription [5-7]. Cytokines are also involved in physiological processes, for example regulating blastocyst implantation during the first trimester of pregnancy [8], as well as placental invasion [9] and tolerance of the fetus [10].

Maternofetal transmission (MFT) of HIV-1 is relatively rare, even in the absence of antiretroviral therapy. R5 HIV-1 isolates are found in most cases of mother-to-child transmission [11-16], and MFT usually occurs during the last trimester [17] pointing to the existence of effective natural control mechanisms particularly during the first months of pregnancy. During the first trimester of pregnancy the maternofetal interface is composed of the placenta (the fetal part) and the maternal uterine mucosa (decidua) [18]. Decidual tissue is defined by its location and function: the decidua basalis is located at the implantation site, in close contact with the placenta, while the decidua parietalis lines the rest of the uterine wall [19]. Blastocyst attachment to the decidua induces placental cell differentiation. A contingent of placental cells, known as extravillous trophoblast cells, invades the decidua during the first trimester of pregnancy.

Immune cells represent a large component of decidual tissue and are composed of natural killer cells (dNK), antigen-presenting cells (dAPC), T lymphocytes (dT) and small percentages of γδ T lymphocytes and NKT cells [20]. These cells interact with one another and with invading trophoblast cells. Trophoblast cells are not permissive to cell-free HIV-1 infection [21,22] but interaction between trophoblast cells and HIV-1-infected cells allows infectious virions to cross the trophoblastic barrier in an *in vitro *model [23]. We have previously shown that first-trimester decidual tissue contains HIV-1 target cells. CD14^+ ^dAPC are the main targets of R5 HIV-1, while decidual T lymphocytes are the main targets of X4 HIV-1 [24]. As MFT is rare during the first trimester of pregnancy, cell-to-cell HIV-1 dissemination at the maternofetal interface appears to be tightly controlled.

The aims of this study were to analyze decidual soluble factors and their role in the regulation of HIV-1 infection at the maternofetal interface.

## Results

### Characterization of the main decidual mononuclear cell populations

**Figure 1:**
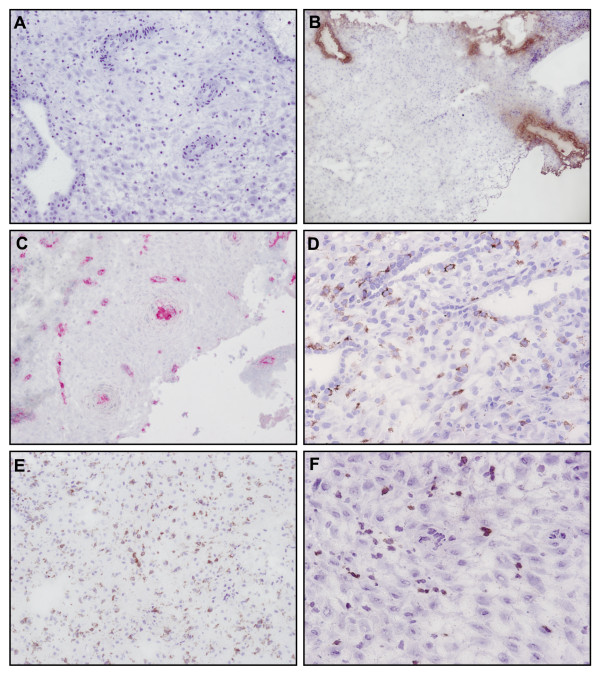
**Characterization of decidual mononuclear cells by immunochemistry**. Frozen decidua basalis sections were stained with Isotype matched Ig control (A), anti-CD34 (B), anti-Cytokeratin 7 (C), anti-CD14 (D), anti-CD56 (E) and anti-CD3 (F). Staining were visualized with diaminobenzidine (brown cells in B, D, E and F) or Vector red (red cells in C) chromogen and tissue sections were counterstained with haematoxylin. Images were taken at ×100 (A, B, C and E) or ×200 (D and F) magnification.

**Figure 2:**
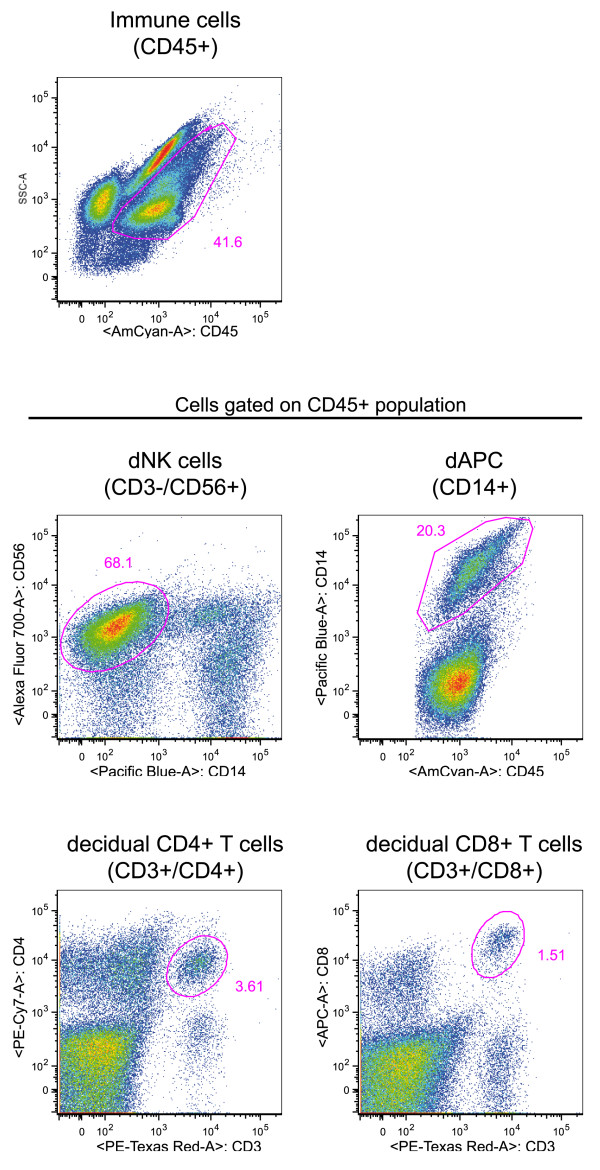
**Analyze of decidual mononuclear cells by flow cytometry**. Cells were gated on the leukocyte population (CD45^+^). Immune cells were identified by expression of surface markers such as CD56 (dNK cells), CD14 (dAPC) and CD3 (dT cells). This experiment is representative of n = 21 decidual samples.

Fresh decidual samples were analyzed by immunohistochemistry. As expected, tissue contained cytokeratin 7^+ ^placental cells and CD34^+ ^endothelial cells. A high number of immune decidual cells were also visualized in isolated tissue (Figure 1); CD56^+ ^NK cells, CD14^+ ^antigen presenting cells and CD3^+ ^T cells. After the digestion of the tissue, mononuclear cells were analyzed by flow cytometry. Immune cell populations present within the decidua are shown on Figure 2 from one representative experiment. As previously described [20,25,26], decidual CD3^-^/CD56^+ ^NK cells represent the main leukocyte population in the decidual tissue (mean 58% ± 7.8). Decidual leukocytes are also composed of CD14^+ ^antigen presenting cells (mean 19% ± 4.7) and CD3^+ ^T lymphocytes (mean 8% ± 5), including CD4^+ ^and CD8^+ ^T lymphocytes (n = 21). Altogether, these results confirmed that the studied tissue was the decidua basalis, a maternal tissue in direct contact with the placenta. Flow cytometry analyzes show that both leukocytes (CD45^+^) and non-leukocytes (CD45^-^) cells were present in decidual mononuclear cells and that dNK cells are the main leukocyte population.

### Decidual culture supernatants contain soluble factors that regulate HIV-1 infection

**Figure 3:**
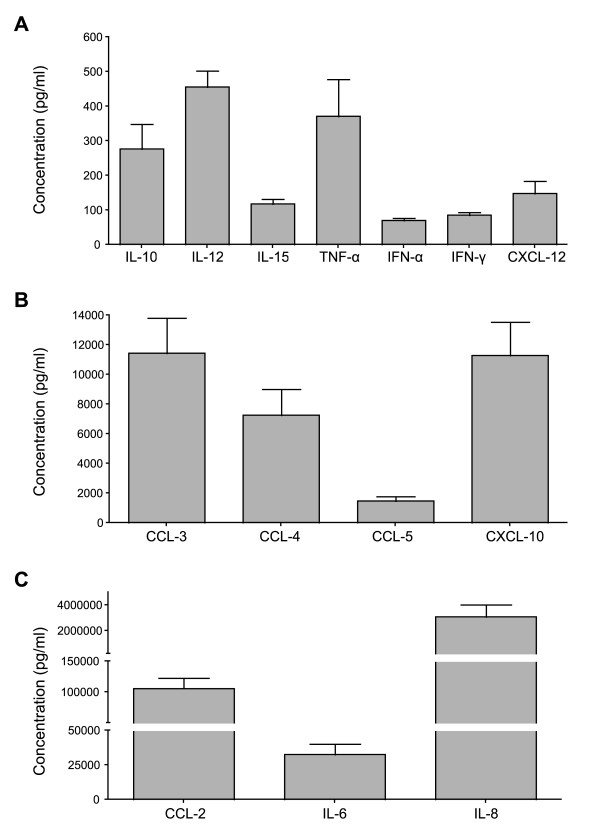
**Cytokine production by decidual mononuclear cells after 24 hours of culture**. Cytokines and chemokines were quantified in 24-hour decidual cell supernatants without any stimulation. Results are expressed in pg/ml, as measured with Luminex technology. Cytokines were classified in 3 groups according to their abundance. Bars represent the mean value of 13 different donors and the error bars indicate the SEM.

To identify soluble factors secreted by decidual cells, we applied Luminex technology and ELISA methods to culture supernatants of decidua basalis mononuclear cells. Cytokines were quantified after 24 hours of culture without stimulation. Figure 3 shows the cytokines detected according to their abundance: low (Figure 3A), medium (Figure 3B), and high (Figure 3C). Cytokines detected at low levels included IL-10 (mean 277 pg/ml ± 72), IL-12 (456 pg/ml ± 46), IL-15 (118 pg/ml ± 14), TNF-α (372 pg/ml ± 107), IFN-α (71 pg/ml ± 6), IFN-γ (86 pg/ml ± 8) and CXCL-12 (148 pg/ml ± 36). Chemokines detected at moderate or high levels included CCL-3 (11460 pg/ml ± 2367), CCL-4 (7272 pg/ml ± 1760), CCL-5 (1492 pg/ml ± 300), CXCL-10 (11300 pg/ml ± 2260) and CCL-2 (106 ng/ml ± 1.7). The pro-inflammatory cytokines IL-6 (33 ng/ml ± 7.6) and IL-8 (3.10^3 ^ng/ml ± 953) were also abundant. The proinflammatory cytokine IL-2 was undetectable (data not show), in keeping with its known absence from the healthy maternofetal interface [27].

The detected soluble factors were also present in similar proportions, but at lower levels in decidua basalis and decidua parietalis histoculture supernatants (data not shown). IL-6 and IL-8 were significantly more abundant in decidua basalis supernantants than in decidua parietalis supernatants (p = 0.0012 and p = 0.01 respectively).

These results showed that decidual cells secreted soluble factors known to regulate HIV-1 infection, including β-chemokines known to inhibit R5 viral entry.

### β-chemokine secretion increases during HIV-1 infection of decidual cells

**Figure 4:**
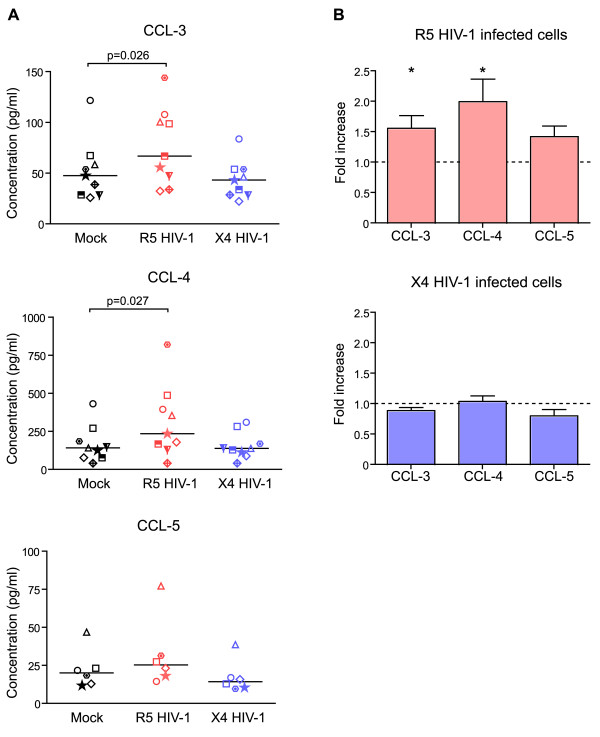
**Modulation of β-chemokine secretion by HIV-1 infection of decidual mononuclear cells**. Decidual mononuclear cells were infected with R5 and X4 HIV-1 (10^-3 ^MOI). (A) β-chemokine secretion was measured 14 days later, after 3 days of culture (day 11 to day 14): uninfected control conditions (black dots), R5 HIV-1 (red dots) and X4 HIV-1 (blue dots). Results are expressed in pg/ml, as measured with Luminex technology. Bars indicate the median values and each donor is represented by a different symbol. Significant changes are indicated. (B) Results are the fold increase in secretion in HIV-1-infected cell culture supernatants compared to uninfected controls. Bars represent the mean of the fold induction and error bars the SEM. At least 6 different donors were used for each experimental condition. Significant changes are indicated by a star (p < 0.05). A one-sample t test was used.

Decidua basalis culture supernatants were analyzed with Luminex technology 14 days after infection, at a time of sustained HIV-1 replication. As the culture medium was renewed every 3 days, reported cytokine levels are those having accumulated between day 11 and day 14. CCL-3 and CCL-4 were significantly more abundant (p = 0.026 and p = 0.027) in the supernatants of R5 HIV-1-infected cells than of non-infected cells. CCL-5 was not significantly more abundant in R5 HIV-1-infected cell supernatants 14 days after infection (p = 0.06)(Figure 4A).

In contrast to R5 HIV-1-infected cells, cytokine secretion was not significantly modulated by X4 HIV-1 infection (Figure 4A and 4B). Production of IL-12, IL-6, CCL-2, CXCL-10 and CXCL-12, that were also detected at day 14, was not significantly affected by either R5 or X4 HIV-1 infection (data not show).

Thus, CCL-3 and CCL-4 release by cultured decidua basalis mononuclear cells was enhanced by R5 HIV-1 infection.

### Decidual CD14^+ ^cells are the main sources of CCL-3 and CCL-4

**Figure 5:**
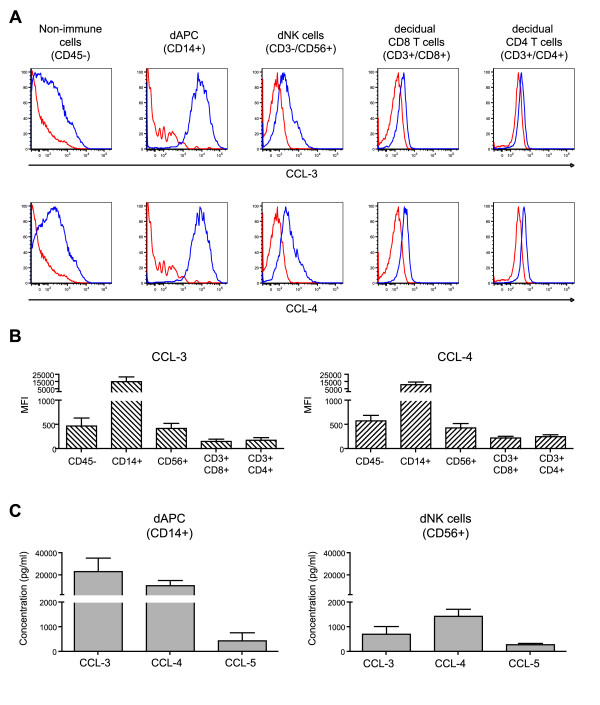
**Identification of β-chemokine-producing cells among decidual mononuclear cells**. (A) Decidual mononuclear cells were cultured for 16 h with Brefeldin A then analyzed by flow cytometry, with gating on the main populations of decidual cells defined by their surface markers. Intracellular staining of CCL-3 and CCL-4 (blue) was analyzed in each cell population and compared to IgG staining (red). Staining for one representative donor is shown. (B) The mean fluorescence indexes (MFI) of β-chemokine staining were obtained for each cell population after subtracting the IgG MFI. All analyses used at least 4 different donors. Bars represent the mean MFI and error bars the SEM. (C) β-chemokines were measured in supernatants of purified dAPC and dNK cells after 3 days of culture, using 10 different donors for dAPC and 7 different donors for dNK. Bars represent the mean and error bars the SEM.

Luminex analysis showed that CCL-3 and CCL-4 release was increased by R5 HIV-1 infection. To identify the source of these chemokines, freshly isolated, HIV-1-uninfected decidua basalis mononuclear cells were analyzed by flow cytometry after intracellular staining. CCL-3 and CCL-4 staining was observed in non leukocytic cells (CD45^-^), natural killer cells (CD56^+ ^dNK), and antigen-presenting cells (CD14^+ ^dAPC) (Figure 5A). The mean fluorescence indexes (MFI) of CCL-3 and CCL-4 in cells from decidua from 4 different women were higher in dAPC than in non leukocytic and dNK cells (Figure 5B). CD4^+ ^T cells and CD8^+ ^T cells both had very low MFIs for CCL-3 and CCL-4.

To confirm the results of flow cytometry, CCL-3 and CCL-4 were quantified by Luminex analysis in 3-day culture supernatants of purified dAPC and dNK cells. Large amounts of both CCL-3 and CCL-4 were detected in dAPC supernatants (23 454 pg/ml ± 12 214 and 10 496 pg/ml ± 4 898, respectively) (Figure 5C). CCL-3 and CCL-4 were also found in dNK cell supernatants, but at lower levels (717 pg/ml ± 314; and 1 445 pg/ml ± 285, respectively). Small amounts of CCL-5 were found in both dAPC and dNK supernatants (452 pg/ml ± 325 and 291 pg/ml ± 50. respectively).

These results showed that dAPC were the main sources of CCL-3 and CCL-4 in the decidua.

### Decidual soluble factors can inhibit HIV-1 infection

**Figure 6:**
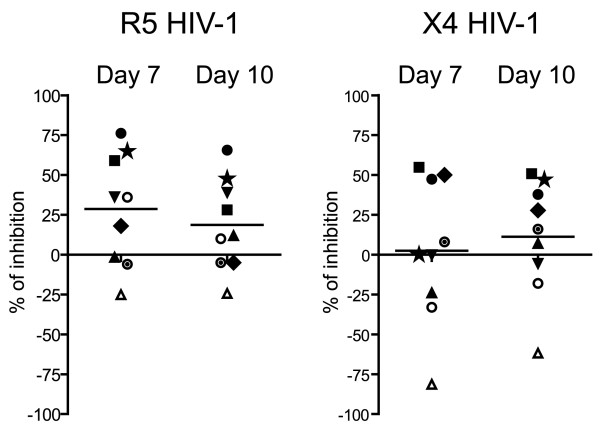
**Inhibition of HIV-1 infection by decidual soluble factors**. Decidual mononuclear cells were cultured for 24 hours without stimulation, then infected with R5 or X4 HIV-1 isolates (10^-4 ^MOI), following with or without a washing step. Viral replication was measured by p24 viral antigen assay in culture supernatants 7 and 10 days post-infection. Results represent the percentage inhibition of HIV-1 infection induced by decidual soluble factors compared with experiments including a washing step. Mean viral p24 levels in culture supernatants were 367 pg/ml at day 7 and 6839 pg/ml at day 10 for R5 HIV-1; and respectively 42 at day 7 and 613 pg/ml at day 10 for X4 HIV-1. Experiments used 9 different donors, each represented by a different symbol, and lines indicate the mean inhibition. Mean inhibition of R5 HIV-1 infection was statistically significant at day 7 (mean 28.64% p = 0.039) but not at day 10 (mean 18.69% p = 0.088). Inhibition of X4 HIV-1 infection observed at day 10 was not statistically significant (mean 11.25% p = 0.375).

To examine the role of the cytokine environment in the inhibition of viral replication, decidua basalis mononuclear cells were cultured for 24 hours before infection with R5 HIV-1 or X4 HIV-1. The cells were then infected, following a washing step or without a washing step (i.e. in the presence or absence of their respective 24-hour supernatants). R5 HIV-1 infection of decidual mononuclear cells was inhibited, as shown by the p24 antigen assay 7 days post-infection, in experiments with 6 out of 9 donors (range 0 to 80%, mean 28.64% ± 11.6; p = 0.039) (Figure 6). Inhibitory activity was lower 10 days post-infection (mean 18.69% ± 9.6; p = 0.088). Inhibition of X4 HIV-1 infection was observed in experiments with 6 out of 9 donors, 10 days post-infection (mean 11.25% ± 11.9; p = 0.375)(Figure 6); however, in contrast to the effect on R5 HIV-1 infection, the mean percentage of inhibition was not statistically significant.

**Figure 7:**
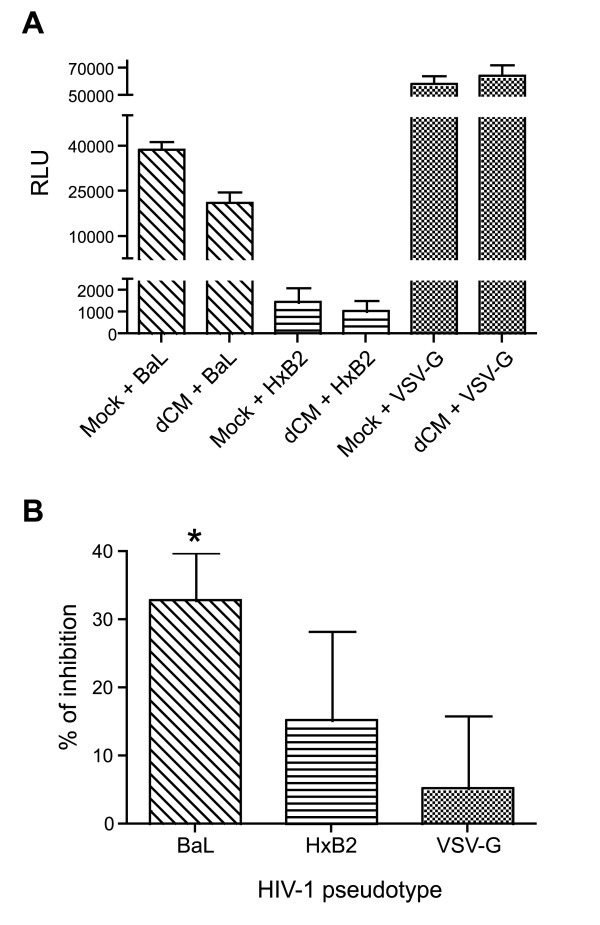
**Decidual soluble factors inhibit the HIV-1 entry step**. HeLa P4P cells were pretreated for 1 hour with fresh medium or 24 h decidual conditioned medium (dCM), and then infected with R5 HIV-1, X4 HIV-1 or VSV-G pseudotypes (6 ng of p24). (A) Luciferase activity was measured 72 h post-infection. A representative experiment is shown. (B) Experiments were performed individually with dCM from at least 7 different donors. Bars indicate the mean inhibition of pseudotype infection; and error bars the SEM. Significant inhibition is represented by a star (p < 0.05). A one-sample t test was used.

To determine whether decidual soluble factors inhibited HIV-1 entry, the HeLa P4P cell line, which expresses the CD4 HIV receptor and also the co-receptors CCR5 and CXCR4, were infected by HIV-1 pseudotypes in the presence or absence of 24 h decidual conditioned medium (dCM). Efficiency of infection was measured in terms of luciferase activity (representative experiment in Figure 7A). dCM from 7 out of 8 donors significantly reduced R5 HIV-1_BaL _pseudotype infection (mean 32.9% ± 7, range 0-58.4%; p = 0.002), while HIV-1_VSV-G _pseudotype infection was unaffected (mean 5.25% ± 10.7, p = 0.640) (Figure 7B). dCM from 5 out of 7 donors reduced X4 HIV-1_HxB2 _pseudotype infection, but not significantly (mean 15.2% ± 13.1, range 0-68%; p = 0.289).

Altogether, these results indicated that decidual culture supernatants participated in the inhibition of HIV-1 entry.

## Discussion

This study suggests that soluble factors secreted by decidual cells can inhibit HIV-1 infection. We first show that decidual mononuclear cells produce soluble factors known to modulate HIV-1 infection. Decidual cells were cultured without exogenous stimulation, contrary to some other studies [28,29]. The types and levels of the cytokines detected in decidual mononuclear cell supernatants were similar to those found in decidual histoculture supernatants (data not show), suggesting that the isolation and culture procedure did not significantly modify the secretion profile. Decidual soluble factors include cytokines known to stimulate HIV-1 infection by promoting inflammation and viral replication including IL-12, TFN-α, IL-6 and IL-8 [6,30,31], but also anti-inflammatory (IL-10) and antiviral cytokines (IFN-α, IFN-γ, CXCL-12, CCL-3, CCL-4 and CCL-5) that inhibit HIV-1 infection [3,4,32,33]. Interestingly, it has been reported that the placenta secretes similar profiles of pro- and anti-inflammatory cytokines to those observed here with the decidua [34,35]. At the maternofetal interface, this pro- and anti-inflammatory balance stimulates leukocyte recruitment and angiogenesis, and regulates placental trophoblast invasion during pregnancy. Moreover, this balance is described in other mucosae such as the gut mucosa, where it is known to regulate microflora tolerance and to prevent pathogen invasion [36].

We have previously shown that decidual mononuclear cells are permissive for HIV-1 infection *in vitro *[24]. Here, we found that secretion of the β-chemokines CCL-3 and CCL-4 was significantly upregulated during R5 HIV-1 infection of decidual mononuclear cells, but not during X4 HIV-1 infection. Some viral proteins, such as Nef, are known to induce β-chemokine production [37]. R5 HIV-1 replicates more efficiently than X4 HIV-1 in decidual cells [24]. The observed increase in β-chemokine secretion during R5 HIV-1 infection could therefore be due to HIV-1 replication. Similar β-chemokine upregulation during HIV-1 infection has also been described in other tissue types, such as lymph nodes and gut-associated lymphoid tissue [38]. This increased β-chemokine secretion was suspected of promoting viral dissemination during the primary infection, through recruitment of immune cells, including HIV-1 target cells [38]. On the other hand, elevated β-chemokine production could also participate in the control of HIV-1 spread, as these soluble factors have been reported to inhibit cell-to-cell HIV-1 infection [23]. Moreover, CCL-3, CCL-4 and CCL-5 are known to inhibit R5 HIV-1 entry by competitively binding CCR5 [3]. β-chemokines might limit the number of infected cells within the decidua and such limit the risk of transmission to the fetus due to the contact of decidual infected cell and placental trophoblast cells. We found that the main source of CCL-3 and CCL-4 in the decidua was CD14^+ ^cells. We detected no CCL-5 production by purified dNK cells or decidual CD14^+ ^cells when using flow cytometry, while small amounts of CCL-5 were detected in both cultures when we used Luminex technology. We have previously shown that dAPC CD14^+ ^cells are the main decidual cell target of R5 HIV-1 [24]. Thus, secretion of HIV-1-inhibiting factors by these cells could constitute a mechanism of autocrine protection, as previously described with peripheral CD4^+ ^T lymphocytes [39]. The latter authors found that CD4^+ ^T lymphocytes, which produce the chemokine CCL-4, were 10 times less infected *in vivo *than cells that did not produce CCL-4.

Decidual soluble factors inhibited R5 HIV-1 infection; and, to a lesser extent, X4 HIV-1 infection, variably from one donor to another. In addition, R5 HIV-1 inhibition was higher at day 7 than at day 10, pointing to an effect on the viral entry step. Similarly, X4 HIV-1 inhibition was weaker at day 14 than at day 10, while no significant replication was noted at day 7. We have previously shown that X4 HIV-1 infects decidual mononuclear cells less efficiently than R5 HIV-1 [24]. To confirm that decidual soluble factors inhibit the viral entry step, we used HIV-1 pseudotypes. Decidual soluble factors inhibited the entry of HIV-1 pseudotypes bearing the HIV-1 (R5 or X4) envelope but not the entry of the VSV-G-bearing HIV-1 pseudotype, which is independent of receptor usage. Inhibitory activity did not correlate directly with the level of CCL-3, CCL-4 or CCL-5, or with the total amount of β-chemokines (data not shown). However, we assayed the chemokines in cell supernatants, and it is conceivable that decidual cells consume a proportion of the β-chemokines they secrete. Furthermore, other decidual soluble factors might also be involved in inhibiting HIV-1 infection, possibly in synergy with β-chemokines. Antimicrobial peptides can inhibit HIV-1 entry in target cells [40-43], and have been detected in human decidua [44] and the female reproductive tract [45,46]. High levels of antimicrobial peptides have been detected in vaginal secretions from HIV-1-exposed but uninfected individuals [47] and have been linked to a low rate of mother-to-child transmission [48].

Inhibition of HIV-1 infection by decidual soluble factors including β-chemokines, appears to constitute a protective mechanism. In view of the large inter-individual differences in the degree of viral inhibition observed here, other mechanisms might contribute to the control of HIV-1 transmission at the maternofetal interface. We found that dNK cells also produced CCL-3, CCL-4 and CCL-5, suggesting that they could have a role in the control of HIV-1 transmission. A recent study has shown that NK cells from non-pregnant uterine mucosa can inhibit X4 HIV-1 infection by secreting high levels of CXCL-12 [49]. NK cells are the main decidual immune cell population [20] and cross-talk with dAPC appears to be crucial for maintenance of pregnancy [50-52]. Interactions between dNK cells and dAPC might stimulate the production of anti-HIV-1 factors and thus enhance autocrine protection of target cells.

## Conclusion

We report that decidual mononuclear cells naturally produce large amounts of chemokines, and that R5 HIV-1 infection significantly enhances production of the β-chemokines CCL-3 and CCL-4. The main source of these chemokines was decidual CD14^+ ^antigen-presenting cells, which are also the main decidual target cell for R5 HIV-1. Furthermore, we provide evidence that decidual soluble factors, including β-chemokines, participate in inhibiting HIV-1 entry in the decidua. These findings also suggest that the first trimester maternofetal interface is a relevant model for studying determinants of natural protection against mucosal HIV-1 infection.

## Materials and methods

### Human decidual tissue

Decidual tissue samples were obtained from healthy women undergoing voluntary termination of pregnancy during the first trimester (6-10 weeks) at Antoine Béclère Hospital, Clamart, France and Tenon Hospital, Paris, France. All the women gave their written informed consent to the use of their tissues. The study was approved by the ethics committee of Hôtel Dieu, Paris, France, the Assistance Publique des Hôpitaux de Paris (n° VAL/2006/06-41/01) and the Biomedical Research Committee of the Institut Pasteur, Paris, France (n° RBM/2005.024).

Histocultures of decidua parietalis and decidua basalis were performed as previously described by Marlin et al [24].

For cell isolation, freshly isolated decidual tissue was minced into small fragments and digested for 1 hour at 37°C under agitation in RPMI 1640 culture medium (Gibco) with 1 mg/ml collagenase IV (Sigma, St Quentin Fallavier, France) and 50 U/ml recombinant DNase I (Roche, Meylan, France). The cell suspension was successively filtered through 100 μm, 70 μm and 40 μm pore-size sterile nylon cell strainers (BD Biosciences, Le pont de Claix, France). The mononuclear cell population was isolated with Lymphocyte Separation Medium (PAA) and cultured in the rich culture media Ham F12: DMEM Glutamax (v:v) (Gibco) supplemented with 15% foetal calf serum (PAA, Les Mureaux, France), penicillin (0.1 U/l) and streptomycin (1 × 10^-8 ^g/l) (Gibco). CD14^+ ^and NK cells were purified with anti-CD14 and anti-CD56 magnetic beads, respectively, as recommended by the manufacturer (Miltenyi, Paris, France).

### HIV-1 primary isolates and pseudotypes

Decidual histocultures and decidual mononuclear cells were infected with two HIV-1 primary isolates, HIV-1_BaL _and HIV-1_LAI _(with R5 or X4 tropism respectively). The isolates were amplified on PHA-stimulated PBMC from two blood donors for 10 days. PBMC cultures were maintained in RPMI 1640 Glutamax (Gibco) supplemented with 10% fetal calf serum, penicillin, streptomycin and 100 U/ml recombinant interleukin-2 (Chiron-Nederlands). Virions were concentrated by centrifugation on Vivaspin 100 000 Kda (Sartorius, Palaiseau, France) at 1400 *g *for 40 minutes.

Luciferase-expressing viral pseudotypes were based on the NL4-3 HIV-1 and VSV-G (amphotropic), BaL (R5) or HxB2 (X4) env plasmids [53-55]. The viral pseudotypes were generated by transfecting HEK-293T cells with the corresponding cDNA plasmid (pNL4-3ΔEnvLuc+ plus the appropriate env cDNA) using the transfection reagent SuperFect transfection reagent (Qiagen) as directed by the manufacturer. The pNL4-3ΔEnvLuc+ lacks the *nef *gene and the resulting pseudotype is non replicative. Supernatants were collected 72 hours after transfection, assayed for the viral pseudotypes by means of p24 antigen ELISA (Zeptometrix) and titrated on HeLa P4P cells. The efficiency of infection by the viral pseudotypes was determined by measuring luciferase expression with Luciferase Reagent (Promega) and a Glomax luminometer.

### Immunohistochemistry

Decidual sections were obtained by embedding freshly isolated decidual tissue in Tissue-Tek (Sakura, Gentaur, Paris, France) and snap-frozen in an isopentane/liquid nitrogen bath. Frozen Tissue-Tek blocks were cut with a cryostat and frozen sections (5 μm thick) were fixed in acetone and rehydrated in TBS (Dako, Trappes, France). Tissue sections were stained for CD14 (RMO52, Beckman Coulter), CD3 (F7.2.38, Dako), CD34 (QbEnd10, Beckman Coulter), CD56 (N901, Beckman Coulter) or Cytokeratin 7 (OV-TL 12/30, Dako). Endogenous peroxidase and alkaline phophatase (AP) were blocked for 10 minutes by addition of hydrogen peroxide and levamisol (Dako). Surface markers were visualized using the Envision+ dual link system (Dako), or using biotin-streptavidin-alkaline phosphatase complex and Vector Red (Abcys, Paris, France), as an AP substrate. Tissue sections were counterstained with haematoxylin (Labonord, templars, France), mounted in permanent medium and analysed with a Nikon Eclipse 80i microscope.

### Cytokine assays

The main cytokines involved in the regulation of HIV-1 infection, namely IL-2, IL-10, IL-12, IL-15, TNF-α, IFN-α, IFN-γ, CCL-3 (MIP1-α), CCL-4 (MIP1-β), CCL-5 (RANTES), IL-6, IL-8, CXCL-10 (IP10) and CCL-2 (MCP1) were measured by Luminex assay (Human Cytokine 25-plex antibody bead kit, Invitrogen Corporation, Carlsbad, California). CXCL-12 (SDF1) was measured with the Human SDF1 Quantikine Immunoassay (R & D System, Minneapolis) in the manufacturer's recommended conditions. The cytokines were measured in supernatants of unstimulated decidual mononuclear cells collected after 24 hours of culture, or after 72 hours in the case of purified dAPC CD14^+ ^and dNK cells, when cytokine concentrations were maximal in the culture supernatants. To compare cytokine production by infected and non-infected decidual mononuclear cells, 3-days of cultured supernatants (day 11 to day 14) were assayed 14 days after infection with or without HIV-1_BaL _or HIV-1_LAI_.

### Identification of cytokine-producing cells

Decidual mononuclear cells were tested for chemokine production by flow cytometry. After isolation, cells were cultured for 16 hours with Brefeldin A (Sigma-Aldrich) at 5 μg/ml, then labelled with anti-CD45-Amcyan, anti-CD14-Pacific Blue, anti-CD4-PE-Cy7, anti-CD56-Alexa 700 (Becton Dickinson), anti-CD3-PE-TexasRed and anti-CD8-APC (Beckman Coulter) before fixation and permeabilisation with the Intraprep reagent (Beckman Coulter). The cells were then labelled with anti-CCL-3-FITC, anti-CCL-4-FITC, anti-CCL-5-FITC (R & D System, Minneapolis) or IgG-FITC (control). Flow cytometry was performed with an LSRII device (Becton Dickinson) and FlowJo 9.0.1 software.

### HIV-1 infection

Decidual mononuclear cells were incubated for 1 hour with HIV-1 isolates or uninfected PBMC supernatant, at 10^-3 ^MOI, then washed 3 times and cultured at 10^6 ^cells/ml. Viral production was measured every 3 or 4 days by detection of p24 antigen in cell culture supernatants by ELISA (Zeptometrix).

### Inhibition of viral production

To examine the influence of the cytokine environment on the inhibition of viral replication, decidual mononuclear cells were cultured for 24 hours before infection with HIV-1_BaL _or HIV-1_LAI_. After 24 hours, the cells were infected with 10^-4 ^MOI for 1 hour at 37°C following or not a washing step. After 3 washes post-infection, the cultured cells were resuspended in culture medium and HIV-1 p24 antigen was titrated by ELISA in the culture supernatants at day 7 and day 10.

### HIV-1 entry inhibition assays

HeLa P4P cells (CD4^+ ^CCR5^+ ^CXCR4^+^) [56] were seeded at 10^4 ^cells/well and cultured for 24 hours in 96-well plates. Entry inhibition assays were performed by incubating the cells for 1 hour with 100 μl of decidual conditioned medium collected after 24 h of decidual mononuclear cell culture, or with fresh culture medium (Mock medium), followed by treatment with 6 ng of p24 equivalent of each HIV pseudotype for 1 hour. The cells were then washed in 1X phosphate buffered saline (PBS) and cultured for 72 hours at 37°C. HeLa P4P cells were washed in 1X PBS and lysed with 100 μl of Cell Lysis Buffer (Promega). The cell lysates (10 μl) were used to determine luciferase activity as described above. Results are expressed as Relative Light Units (RLU)/100 μl of lysate. Each experimental condition was performed in triplicate.

### Statistical analysis

Each condition was tested in at least four independent experiments. The one-sample t test was used. The hypothetical value was 1 for the fold-change graph and 0 for the percentage inhibition graph. Significance was assumed at p < 0.05. Prism 4 software (Graph Pad) was used for all analyses.

## Conflict of interests

The authors declare that they have no competing interests.

## Authors' contributions

Conceived and designed the experiments: RM MTN FBS EM. Performed the experiments: RM MTN MD CC. Analyzed the data: RM MTN FBS EM. Contributed to materials: ALB NB. Wrote the paper: RM MTN MD EM. All authors read and approved the final manuscript.
